# Cancer-testis antigen cyclin A1 is broadly expressed in ovarian cancer and is associated with prolonged time to tumor progression after platinum-based therapy

**DOI:** 10.1186/s12885-015-1824-6

**Published:** 2015-10-24

**Authors:** Ruza Arsenic, Elena Ilona Braicu, Anne Letsch, Manfred Dietel, Jalid Sehouli, Ulrich Keilholz, Sebastian Ochsenreither

**Affiliations:** 1 Department of Pathology, Institute of Pathology, Charité – University Hospital Berlin, 10117 Berlin, Germany; 2 Departement of Gynecology, University Hospital Berlin, 13353 Berlin, Germany; 3 Department of Hematology, Oncology and Tumor Immunology – University Hospital Berlin, 12200 Berlin, Germany; 4 Charité Cancer Comprehensive Center, Charité, 10117 Berlin Germany

**Keywords:** Immunotherapy, Ovarian cancer, Cytotoxic T-lymphocytes, Cyclin A1

## Abstract

**Background:**

Cyclin A1 is essential for male gametopoiesis. In acute myeloid leukemia, it acts as a leukemia-associated antigen. Cyclin A1 expression has been reported in several epithelial malignancies, including testicular, endometrial, and epithelial ovarian cancer (EOC). We analyzed Cyclin A1 expression in EOC and its correlation with clinical features to evaluate Cyclin A1 as a T-cell target in EOC.

**Methods:**

Cyclin A1 mRNA expression in EOC and healthy tissues was quantified by microarray analysis and quantitative real-time PCR (qRT-PCR). Protein expression in clinical samples was assessed by immunohistochemistry (IHC) and was correlated to clinical features.

**Results:**

Cyclin A1 protein was homogeneously expressed in 43 of 62 grade 3 tumor samples and in 1 of 10 grade 2 specimens (*p* < 0.001). Survival analysis showed longer time to progression (TTP) among patients with at least moderate Cyclin A1 expression (univariate: *p* = 0.018, multivariate: *p* = 0.035). FIGO stage, grading, age, macroscopic residual tumor after debulking, and peritoneal carcinomatosis / distant metastasis had no impact on TTP or overall survival (OS).

**Conclusion:**

Cyclin A1 is highly expressed in most EOCs. The mechanism behind the prolonged TTP in patients with high Cyclin A1 expression warrants further investigation. The frequent, selectively high expression of Cyclin A1 in EOC makes it a promising target for T-cell therapies.

**Electronic supplementary material:**

The online version of this article (doi:10.1186/s12885-015-1824-6) contains supplementary material, which is available to authorized users.

## Background

Epithelial ovarian cancer (EOC) is the seventh most common cancer and the eight most common cause of cancer-related death among women worldwide [1], with high-grade serous carcinoma being the most common histology [2]. About two-thirds of patients with EOC are diagnosed at an advanced stage with peritoneal or visceral spread [3]. Standard treatment in that setting is cytoreductive surgery followed by chemotherapy with platinum and paclitaxel. Despite high response rates to first-line systemic treatment, all patients with initially advanced or secondary metastatic disease relapse, develop platinum resistance, and eventually die from the disease [4]. Recently, systemic treatment was improved by the addition of new agents (e.g., bevacizumab and PARP inhibitors) to the classical cytostatic therapy. Nevertheless, there is still an unmet need for therapeutic modalities that can contribute to more sustainable tumor control without constant exposure to treatment-related toxicity.

Targeted T-cell therapy consisting of vaccination or the adoptive transfer of T-cells against defined tumor-associated antigens (TAA) is a reasonable extension of established treatment strategies.

EOCs are immunogenic tumors with spontaneous T-cell responses in more than 50 % of patients [5–7]. While the presence of tumor-infiltrating intraepithelial lymphocytes is associated with prolonged progression-free survival (PFS) and overall survival (OS), immune evasive factors, such as the expansion of regulatory T-cells or the expression of PD-L1 and endothelin B receptor, correlate with poor survival [8, 9]. Patients with advanced stage EOC after initial debulking and cytostatic treatment are excellent candidates for targeted T-cell therapy because of their minimal tumor burden and tumor immunogenicity, which may be enhanced by previous paclitaxel treatment [5–7].

One essential step in the development of a T-cell based therapy is the choice of an appropriate antigen [10, 11]. Besides the so-called neoantigens, which are generated by somatic mutations in the neoplastic cells (e.g., p53) and are usually patient-specific, the targetable TAAs in EOC are usually self-antigens, which are non-mutated proteins aberrantly expressed by the tumor. More than 20 self-antigens have been described in EOC, including several membrane-bound proteins with limited processing and presentation (e.g., ERBB2, MUC16, and Mesothelin) [12] and others that are significantly expressed in normal tissue (e.g., Mesothelin, Cyclin I, FOLR1, WT1, and MUC1)., implying not only tolerance by the peripheral T-cell repertoire, but also the risk of immunogenic toxicity (on-target/off-tumor toxicity) in the case of an effective T-cell response. The expression of some TAAs is irrelevant for the maintenance of the malignant phenotype, with unstable expression in the malignant cells (e.g., MUC16). Further, some TAAs are only expressed in a small percentage of patients (e.g., ERBB2), are heterogeneously expressed (e.g., NY-ESO-1), or are expressed in the activated T-cells (e.g., Survivin, hTERT) [13-18]. Therefore, the identification of new TAAs with stable, homogeneous, and selective expression in EOC is an urgent need for the development of T-cell-based therapies for EOC.

We recently described Cyclin A1 as a T-cell antigen with aberrant expression in the stem cell compartment of acute myeloid leukemia [19]. In healthy individuals, Cyclin A1 expression is restricted to the testis, where it plays a crucial role in meiosis I of gametopoiesis. The highly selective expression pattern has not only been shown at the mRNA and protein level, but also by ligandome analysis, demonstrating that Cyclin A1 peptides bind to MHC class I in acute myeloid leukemia cells but not in healthy tissues or during normal hematopoiesis [10, 19]. Cyclin A1 proved to be immunogenic in vitro, and several MHC class I epitopes have been described. In an in silico analysis of Cyclin A1 expression in solid tumors, we found high Cyclin A1 expression in all four specimens analyzed. Currently, we have only sparse data on the impact of Cyclin A1 on proliferation, invasiveness, and resistance to apoptosis in EOC [20]. Furthermore, the potential prognostic impact of Cyclin A1 expression in EOC has not yet been addressed.

The aim of this study was to analyze Cyclin A1 expression at both the mRNA level and the protein level with regard to the potential use of Cyclin A1 as a T-cell antigen in EOC. Furthermore, correlations with histopathological and clinical features were performed to investigate the possible prognostic impact of Cyclin A1 expression in EOC.

## Methods

### Patients and specimens

Microarray data sets were obtained from the NCBI GEO database. For qRT-PCR and IHC, 72 patients were selected from the ‘Tumor Bank Ovarian Cancer Network’ database based on histology and initial treatment. The tumor specimens were collected before the onset of the chemotherapy. All patients suffered from serous EOC and received cytoreductive surgery followed by platinum-based chemotherapy. Patients provided written informed consent for use of their biomaterial samples in biomarker studies. Consent was obtained using the standardized informed consent forms of the participating institutions. The project and consent process was approved by the ethic board of the Charité Hospital, Berlin (reference number EA2/005/14).

### Microarray data analysis

To further determine the frequency of Cyclin A1 expression in EOC, 20 tumor samples (GSE14001) were analyzed along with healthy tissues (GSE3526). The samples were normalized using the invariant set method (dChip 2.0 software) [21]. Samples exceeding the mean expression level plus three standard deviations of the healthy, non-testicular tissue samples were considered positive (Additional file 1).

### Quantitative real-time PCR

Total RNA was extracted from cells and frozen tissue using Trizol reagent (Invitrogen, Carlsbad, California, USA) and from paraffin-embedded samples using the RNeasy FFPE kit (Qiagen,Venlo, Niederlande). The RNA was reverse transcribed using Superscript III (Invitrogen). Complementary DNA from healthy tissues was obtained from Clontech (Mountain View, CA, USA). Quantitative real-time PCR (qRT-PCR) was performed on a Light Cycler instrument (Roche, Basel, Switzerland) with an annealing temperature of 60 °C using previously published primers and probes [19]. Crossing points were plotted against the standard curves of pCR4-TOPO plasmids (Invitrogen) containing the respective PCR products. All reactions were performed in duplicate. Expression levels were presented as copies of Cyclin A1 per copies of the housekeeping gene *GAPDH*. Samples exceeding the mean expression level plus three standard deviations of the samples of healthy tissues were considered positive.

### Immunohistochemistry staining

Tumor specimens were cut in 4-μm-thick sections and mounted on glass slides. After paraffin removal, hydration, heat-activated antigen retrieval in the DAKO-PT-link module (DAKO Glostrup, Denmark), and blocking of endogenous peroxidase activity by exposure to 3 % hydrogen peroxide for 20 min, the slides were incubated at 4 °C overnight with mouse anti-Cyclin A1 monoclonal antibody, clone 722407 (R&D Systems, Abingdon, UK) at a 1:25 dilution. After washing, the sections were processed with a Polymer HRP detection system (PV-9000, Zhongsam Company, Beijing, China). The slides were than stained with 3,3′-Diaminobenzidine and counterstained in hematoxylin. The staining intensity and the percentage of positive cells were evaluated at 400× magnification without knowledge of clinical data.

Only cells with nuclear Cyclin A1 staining were considered positive. The staining intensities were expressed as weak (1), weak to moderate (1.5), moderate (2), moderate to strong (2.5), or strong (3). The evaluation was performed by an experienced gynecopathologist (RA).

### Statistics

A non-parametric correlation analysis was performed by calculating Spearman’s ρ. Expression values were compared using a two-tailed Mann–Whitney test or a Kruskal-Wallis test. TTP and OS were calculated from the time of initial surgery. Survival analysis was performed using a log-rank test. A multivariate survival analysis was performed using Cox regression. Statistical analyses were conducted using SPSS 19 statistical software (SPSS Inc., Chicago, IL, USA).

## Results

### Patients

Cyclin A1 expression was analyzed immunohistochemically in tumor material from 72 patients primarily with advanced EOC who underwent cytoreductive surgery followed by platinum-based chemotherapy (carboplatinum/paclitaxel in 71 patients, cisplatinum/paclitaxel in one patient). The mean age of the patients was 59 (range: 37 to 78) years. Further patient characteristics are given in Table 1.

**Table 1:** Clinico-pathological characteristics of the patients

	*n*	Percent
Grade		
2	10	14
3	62	86
Stage (FIGO)		
II	3	4
III	59	82
IV	10	14
Peritoneal carcinomatosis		
No	8	11
Yes	64	89
Residual macroscopic tumor rest		
No	52	72
Yes	20	28
Primary platinum sensitivity*		
Sensitive	48	67
Resistant	24	33

*Platinum sensitivity is defined as relapse-free survival for at least six months after the end of initial platinum-based chemotherapy

### Cyclin A1 is homogenously expressed in most high-grade epithelial ovarian cancers

To identify tumor entities with frequent aberrant Cyclin A1 expression, a microarray panel from the NCBI GEO data base (GEO, http://www.ncbi.nlm.nih.gov/geo/) containing healthy tissues and samples of 21 tumor entities was screened. Probe set 205899_at, which represents Cyclin A1 on the respective microarray platform, and which has been validated in earlier studies, was analyzed [19].

The panel contained four EOC samples, which all showed significant overexpression of Cyclin A1 compared to healthy tissues (data not shown).

**Fig. 1:**
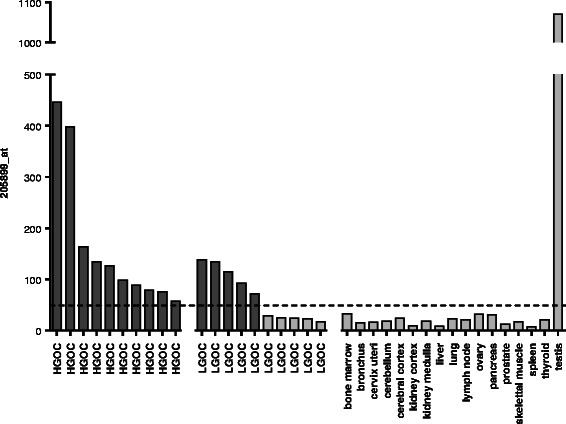
Microarray data showing high Cyclin A1 expression in high-grade and low-grade serous ovarian carcinoma and testis relative to other tissues. Graph shows model-based expression of probe set 205889_at, representing Cyclin A1. LGOC, low-grade ovarian cancer; HGOC, high-grade ovarian cancer. Mean value + 3SD is marked by the horizontal bar

**Fig. 2:**
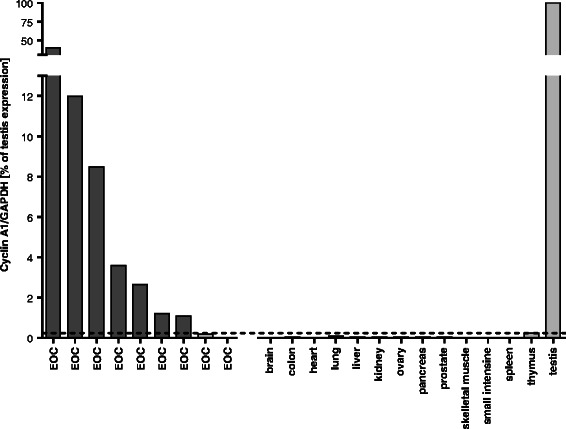
qRT-PCR of snap-frozen EOC specimens and of cDNA from healthy tissues, showing high Cyclin A1 expression in testis and seven of the nine EOCs. Graph shows expression of Cyclin A1 (copies/copies of *GAPDH*) in relation to expression in testis (=100 %). Mean value + 3SD is marked by the horizontal bar

Next, a microarray containing a panel of 20 EOC specimens was analyzed and revealed Cyclin A1 expression in all ten high-grade samples and in five of the ten low-grade samples (GSE14001, GSE3526, Fig. 1). To further validate the in silico findings, Cyclin A1 was quantified by qRT-PCR in eight snap-frozen EOC specimens and in a variety of healthy tissues. Again, there was no Cyclin A1 expression in healthy tissues except for the testis, but seven of the nine EOC specimens tested positive for Cyclin A1 mRNA (Fig. 2).

**Fig. 3:**
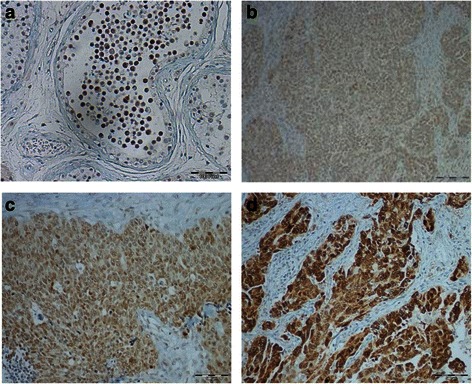
Immunohistochemistry for Cyclin A1. **a** Positive control testis. **b**–**d** Representative immunohistochemical staining in serous carcinoma of the ovary: B-weak staining intensity; C-moderate staining intensity; D-strong staining intensity. Original magnification 20 × 10

**Fig. 4:**
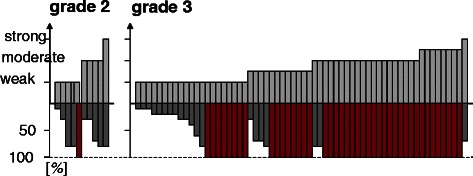
Immunohistochemical Cyclin A1 expression features (staining intensity and percentage of positive cells) of all 72 specimens analyzed depending on histopathological grade. Homogenous positivity (shaded in red) in 43 of 62 grade 3 specimens, but in only one grade 2 specimen

**Fig. 5:**
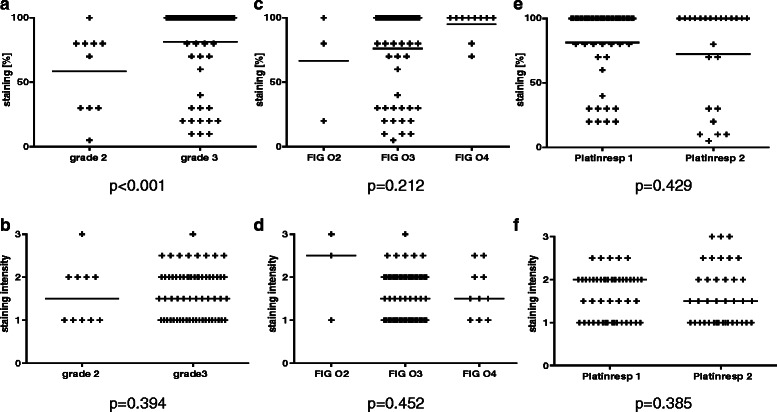
Comparison of staining intensities (**b**,**d**,**f**) and percentage of positive cells (**a**,**c**,**e**) depending on histopathological grading (**a**,**b**), FIGO stage (**c**,**d**), and platinum responsiveness (**e**,**f**). * indicates significant differences (non-parametric). Median values are marked by horizontal bars

Cyclin A1 was then analyzed at the protein level to confirm proper translation and to detect potential heterogeneity of expression within the tumors. To validate the immunohistochemical staining, RNA was extracted from paraffin-embedded slides of nine samples, and qRT-PCR was performed. There was a significant correlation between staining intensity and Cyclin A1 mRNA expression (ρ = 0.685, *p* = 0.042, data not shown), confirming the specificity of the immunohistochemical staining. Representative images of varying staining intensities are shown in Fig. 3. There was a strong correlation between the staining intensity and the percentage of positive cells in all 72 patients (ρ = 0.436, *p* = 0.0001, data not shown). Homogenous Cyclin A1 positivity was observed in 43 of 62 grade 3 specimens, but in only 1 of 10 grade 2 specimens (*p* = 0.005, Fig. 4). The percentage of positive cells, but not staining intensity, was significantly higher in the grade 3 specimens (*p* < 0.001; *p* = 0.394) (Fig. 5 A,B).

### Cyclin A1 expression is associated with prolonged time to progression

**Fig. 6:**
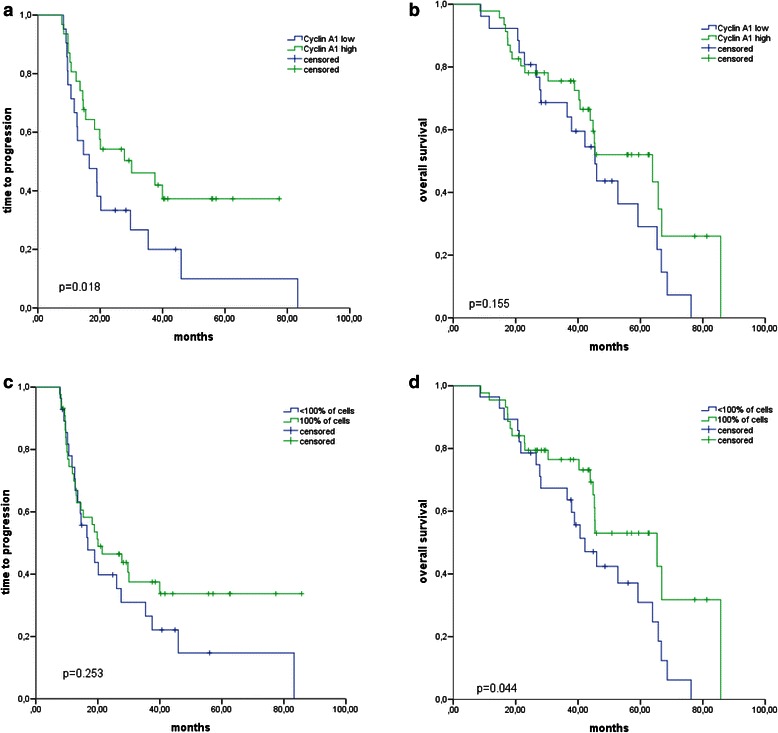
**a** Difference in time to progression after surgery in relation to staining intensity: blue-Cyclin A1^low^; green-Cyclin A1^high^ (*p* = 0.018). **b** Difference in overall survival after surgery in relation to staining intensity: blue-Cyclin A1^low^; green-Cyclin A1^high^ (*p* = 0.155). **c** Difference in time to progression after surgery in relation to percentage of cells: blue- < 100 % of cells; green-100 % of cells (*p* = 0.253). **d** Difference in overall survival after surgery in relation to percentage of cells: blue- < 100 % of cells; green-100 % of cells (*p* = 0.044). The time is given in months

Cyclin A1 expression in all 72 patients was then correlated to the clinical features to identify a potential prognostic relevance of Cyclin A1 in EOC. Median TTP and OS of all patients were 19.0 months (range 7.7 to 85.7) and 46.0 months (8.6 to 85.7), respectively. There were no statistically significant differences in either the intensity of Cyclin A1 staining or the percentage of Cyclin A1-positive cells in regard to the clinical stage, the age at first diagnosis, or presentation with peritoneal carcinomatosis / distant metastasis or platinum sensitivity (Fig. 5 C-F and data not shown). However, high Cyclin A1 expression (Cyclin A1^high^) was associated with prolonged TTP in an univariate survival analysis (*p* = 0.018, 27.5 vs. 14.6 months) (Fig. 6, Additional file 2: Table S1).

**Fig. 7:**
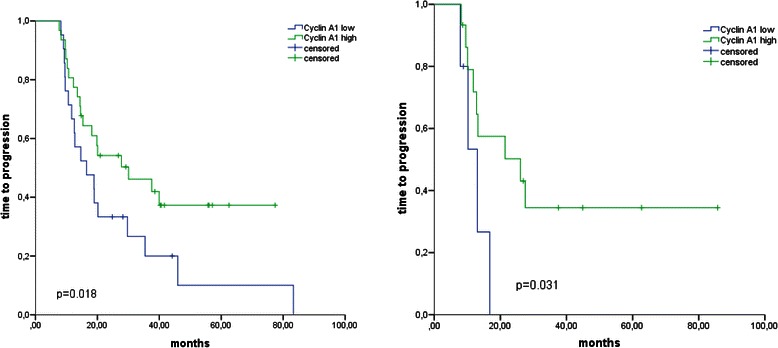
**a** Difference in time to progression after surgery in relation to staining intensity by the patients without macroscopic residual tumor: blue-Cyclin A1^low^ green-Cyclin A1^high^ (*p* = 0.018). **b** Difference in time to progression after surgery in relation to staining intensity by the patients with macroscopic residual tumor: blue-Cyclin A1^low^; green-Cyclin A1^high^ (*p* = 0.031)

To rule out a potential bias from different efficacies in surgical cytoreduction, a second analysis was performed including only patients with residual macroscopic tumor (*n* = 20). In that population, the difference in TTP between Cyclin A1^high^ and Cyclin A1^low^ patients was even greater (median TTP 26.1 vs. 13.0 months), suggesting that Cyclin A1 expression is predictive of patient responsiveness to the standard fist-line chemotherapy regimen (Fig. 7).

To further confirm that observation, we used the online-accessible tool ‘Kaplan-Meier-Plotter’ [22] to analyze data from six independent studies and ‘The Cancer Genome Atlas’ (TCGA, version 2011). Altogether, 264 cases with serous EOC stage II to IV, suboptimal debulking, and platinum-containing treatment were selected. Again, higher Cyclin A1 expression levels were associated with longer TTP (*p* = 0.0088, Additional file 3: Figure S1), while no statistical difference in TTP could be observed in the cohort of patients with optimally debulked EOC (data not shown).

We then conducted a multivariate analysis of TTP including the four covariates with the lowest *p*-values in the univariate analysis: Cyclin A1 staining intensity, the percentage of Cyclin A1-positive cells, FIGO stage, and peritoneal carcinomatosis. Only Cyclin A1^high^ staining intensity was an independent indicator for prolonged TTP (*p* = 0.035) (Additional file 2: Table S1). Furthermore, while homogeneous positivity for Cyclin A1 was associated with longer OS in the univariate analysis (*p* = 0.044, 65.3 vs. 42.2 months) (Fig. 6D), none of the parameters were independent prognostic markers for OS (Additional file 2: Table S1).

### The impact of Cyclin A1 expression on TTP is not associated with cancer molecular subtypes

Recently, four molecular subgroups of high-grade ovarian cancer [C1 (high stromal response), C2 (high immune signature), C4 (low stromal response), and C5 (mesenchymal)] have been described based on microarray data [23]. The most common subtype, C1, is characterized by shorter progression-free survival after initial treatment compared with the other subtypes. To test whether a low Cyclin A1 level could be a surrogate marker for the C1 subtype rather than an independent prognostic factor, the original microarray data was analyzed for Cyclin A1 expression (probe set 205889_at, Additional file 4: Figure S2). Cyclin A1 expression was significantly different among the four subtypes (*p* = 0.001), with C1 showing the highest expression and C5 showing the lowest expression. If Cyclin A1 was a pure surrogate marker for C1, we would have expected a lower expression in C1 in relation to other subtypes. Even though the comparison of our data with mRNA data from a second independent cohort has limited informative value, this observation implies that the impact of Cyclin A1 expression on TTP is not directly associated with the molecular subtype.

## Discussion

Here, we provided the first linear mRNA expression analysis of Cyclin A1 in EOC and healthy tissuesand put immunohistochemical Cyclin A1 expression into a clinical context. We showed that Cyclin A1 is highly and homogeneously expressed in high-grade EOC in a high percentage of patients. Furthermore, we identified Cyclin A1 as an indicator for prolonged TTP after platinum-based first-line chemotherapy, independent of other clinical features, histological grading, and molecular subtype.

The identification of an appropriate antigen that is selectively overexpressed in a given tumor entity is a critical step in the development of a T-cell-based treatment. We pursued a reverse strategy by first selecting the testis-selective antigen Cyclin A1 [19] and then screening its expression in a multitude of different non-hematological tumor entities. In the initial in silico screening, only EOC showed high Cyclin A1 expression in all data sets analyzed. Cyclin A1 overexpression has already been described in a small set of EOC samples as well as in testicular germ cell tumors and endometrial cancer [24]. We decided to pursue Cyclin A1 as a potential T-cell target in EOC for several reasons. First, EOC is considered an immunogenic tumor with T-cell infiltration being associated with better prognosis [6, 8].

Second, initial exposure to paclitaxel seems to enhance the presentation of TAA epitopes by the malignant cells [25, 26] Furthermore, the clinical course of EOC is usually characterized by effective initial tumor reduction by surgery and systemic treatment, followed by an interval without cytostatic therapy and minimal tumor burden, a clinical setting which is considered an ideal condition for immunotherapeutic intervention.

A National Cancer Institute pilot project to prioritize cancer antigens developed a list of weighted “ideal” antigen criteria/characteristics, which can be used to evaluate new tumor antigen candidates. The absence of relevant expression in healthy tissues with the exception of testis, which is considered to be an immunoprivileged site, is one of the central features of a T-cell antigen because of potential on-target/off-tumor toxicities when the antigen is expressed in healthy tissues. This selective oncofetal expression pattern was demonstrated for Cyclin A1 in a previous study [19]. Other criteria, which are not specific to the targeted tumor entity and apply to Cyclin A1, are an intracellular location and a high number of available epitopes. Furthermore, high expression in the tumor in a large fraction of patients is essential. In the larger set of ovarian cancer samples, we detected Cyclin A1 staining in all the samples, with at least moderate staining in half of the samples irrespective of the histological grade. This high frequency of antigen positivity promises easy patient accrual for potential clinical trials and would guarantee broad applicability if Cyclin A1 can be effectively targeted in a clinical setting. Another feature of a suitable antigen is a high frequency of positive cells within the tumor tissue [27]. Cyclin A1 fulfills this criterion, being homogenously expressed at a moderate to high level in more than half of the high-grade carcinomas analyzed. Our mRNA and protein expression data are complemented by the results of a large human leukocyte antigen (HLA)-ligandome study showing Cyclin A1 peptide presentation in the context of MHC class I in ovarian cancer but not in healthy tissues or hematopoiesis (Heiko Schuster, Tübingen, personal communication), making Cyclin A1 an attractive T-cell target in patients with EOC.

The function of Cyclin A1 in normal and malignant somatic cells is only partially understood and might depend on the expression level, differentiation grade, and tissue of origin. Cyclin A1 expression enhances G1/S transition in somatic cells and is associated with enhanced proliferation and invasiveness in cancers of the breast, prostate, urothelium, and thyroid [28-32]. At the same time, the induction of apoptosis by both intrinsic and extrinsic pathways increases the Cyclin A1 protein level by both p53-mediated transcription and post-translational modification. Moreover, Cyclin A1 seems to enhance the pro-apoptotic effect of p53 [20, 33], and the Cyclin A1/CDK2-mediated phosphorylation of p53 enables stable complex formation with topoisomerase I, thereby causing hyper-recombination in p53 mutant cells [34]. Although the data on EOC are very limited, this mechanism might play a significant role in oncogenesis in EOC, given that Cyclin A1 expression was uniformly high in the ovarian cancer samples, and both p53 mutations and genomic instability are characteristic features of EOC [35, 36].

We identified Cyclin A1 expression as a predictive marker for longer TTP after first-line cytostatic treatment in two independent data sets, one based on protein expression and one based on mRNA levels [22]. This effect was independent of the disease stage at first diagnosis, peritoneal carcinomatosis, histological grade and age, and was stronger in patients with suboptimal surgical cytoreduction. The longer TTP in patients with higher Cyclin A1 levels might therefore reflect responsiveness to cytostatic treatment rather than an association between more aggressive tumor biology and later-stage disease at first diagnosis. Cyclin A1 directly interacts not only with p53 but also with at least two members of the Retinoblastoma gene product (pRB) pathway, pRB and E2F-1, which regulates proliferation and is itself modulated by p53 [37]. Several molecules in that complex network (p53, p21, pRb, and E2F-1) have been discussed as predictive markers for treatment response or prognosis in EOC [38-41].

It remains unclear whether Cyclin A1 expression is a surrogate marker for dysregulation of the pRB-p53 network or whether its own anti-apoptotic effect or modulation of the pRB pathway substantially contributes to the longer TTP. p53 is mutated and believed to be transcriptionally defective in the majority of patients. No difference in Cyclin A1 expression could be detected between EOC with wild type p53 and with mutated p53 [42]. This, and the fact that several oncogenes have been identified as Cyclin A1 transcription factors in malignant cells [43, 44], makes a direct Cyclin A1-mediated effect seem more likely.

Gene expression profiling for class discovery has been widely applied to ovarian cancer. The definition of four distinct molecular subtypes of EOC by Tothill et al. has found broad acceptance due to its biological consistency and clinical relevance. Compared with the other subtypes, subtype C1 (high stromal response) has a significantly shorter TTP [23]. To determine whether Cyclin A1 is expressed differentially in the different subgroups, and more specifically, whether a low Cyclin A1 level might be a surrogate marker for the C1 subtype, we compared Cyclin A1 expression among the different subtypes. Cyclin A1 expression was indeed significantly different between the four subtypes, but with expression in C1 significantly higher compared with the other subtypes. When evaluating self-proteins as potential T-cell targets, the actual translation of the protein is a pivotal factor. Consequently, we analyzed our clinical samples with IHC. On the other hand, the assignment to a molecular subtype requires the mRNA expression data of a microarray. Because microarray data of the clinical samples was not available, and Tothill et al. did not provide protein data for Cyclin A1, a direct comparison between the two factors (cluster analysis by microarray and protein expression) was not possible. Despite these limitations, and given that C1 is characterized by short TTP, its association with high Cyclin A1 expression on mRNA level implies that Cyclin A1 is not a pure surrogate maker for C1, and its impact on TTP might be at least partially independent of the molecular subtype.

## Conclusions

Cyclin A1 appears to be a highly suitable antigen in patients with EOC for targeted T-cell therapy because of its selectively high expression in the vast majority of high-grade ovarian cancers irrespective of clinical stage. In view of its predescribed immunological features and expression pattern in EOC, Cyclin A1 should be pursued further as a T-cell target for an application in a clinical setting. Independent of its potential role as a target for T-cell therapy, Cyclin A1 acts as predictive marker for response to standard platinum-based cytostatic therapy, translating into prolonged TTP. This, its differential expression in the molecular subtypes, and the already known interactions with other cell cycle regulating genes indicate a clinically relevant pathophysiological function for Cyclin A1 in EOC, which remains to be further elucidated.

## Additional files

Additional file 1: To identify Cyclin A1-expressing solid cancer entities, HG U133 Plus 2.0 microarray data sets (Affymetrix, Santa Clara, CA) of healthy tissues and tumor samples from the NCBI GEO server were screened (NCBI GEO, http://www.ncbi.nlm.nih.gov/geo/, GSE****). (DOC 20 kb)
Additional file 2: **Table S1.** Cox regression analysis for TTP and OS. Covariates other than Cyclin A1 staining intensity and percentage of Cyclin A1-positive cells for univariate analysis were chosen based on clinical relevance as described in earlier studies [9, 41, 42]. The four covariates with the highest *p*-values were analyzed in a multivariate Cox regression analysis [the number of 49 (TTP) and 41 (OS) events allows a maximum of four covariates]. *b*_*i*_: regression coefficient, CI: confidence interval, HR: hazard ratio. (DOCX 14 kb)
Additional file 3: **Figure S1.** Survival plot depicting the impact of Cyclin A1 expression (Affymetrix probe set 205899_at) on progression-free survival in the patient group with suboptimal debulking and platinum-based therapy using an online-accessible tool (www.kmplot.com/), database version 2015 [*n* = 1648]. Case selection [*n* = 264]: survival: PFS, split patients by median; restrictions: FIGO II, III, IV; histology: serous; debulk: suboptimal; chemotherapy: contains platinum. Log-rank *p* = 0.0088. (PPTX 84 kb)
Additional file 4: **Figure S2.** Differences in Cyclin A1 expression between molecular tumor subtypes (1, 2, 4, and 5), according to the molecular classification of EOC by Tothill et al. Data sets were retrieved from the NCBI GEO database and normalized using the invariant set method (dChip 2.0 software) [23]. C1 showed significantly higher expression and C5 showed significantly lower expression (Kruskall Wallis; *p* = 0.001). Mean value + 3SD is marked by the horizontal bar. (PPT 147 kb)
