# Supplementary material for: Cancer-testis antigen cyclin A1 is broadly expressed in ovarian cancer and is associated with prolonged time to tumor progression after platinum-based therapy
Source: BMC Cancer. 2015 Oct 24;15:784. doi: 10.1186/s12885-015-1824-6 (PMC4619521; doi:10.1186/s12885-015-1824-6)
Supplement: Additional file 1: — To identify Cyclin A1-expressing solid cancer entities, HG U133 Plus 2.0 microarray data sets (Affymetrix, Santa Clara, CA) of healthy tissues and tumor samples from the NCBI GEO server were screened (NCBI GEO, http://www.ncbi.nlm.nih.gov/geo/, GSE****). (DOC 20 kb) [file 12885_2015_1824_MOESM1_ESM.doc]

**Microarray data analysis**

To identify Cyclin A1 expressing entities of solid cancer, HG U133 Plus 2.0 microarray data sets (Affymetrix, Santa Clara, CA) of healthy tissues and tumor samples from the NCBI GEO server were screened (NCBI GEO access numbers in supplemental material).

***Supplemental material:***

NCBI GEO data sets of solid tumors and healthy tissues for screening of Cyclin A1-expressing tumor entities:

GSM134587, GSM134689, GSM134698, GSM134701, GSM143550, GSM143551, GSM155646, GSM155652, GSM155675, GSM155678, GSM183219, GSM183235, GSM183272, GSM183304, GSM242823, GSM242824, GSM258551, GSM258580, GSM281291, GSM281294, GSM281341, GSM281342, GSM305355, GSM305358, GSM310012, GSM310015, GSM324062, GSM324063, GSM350423, GSM350432, GSM350579, GSM350582, GSM350609, GSM350610, GSM371114, GSM371115, GSM408899, GSM408900, GSM414972, GSM484715, GSM484720, GSM80576, GSM80582, GSM80602, GSM80615, GSM80619, GSM80653, GSM80689, GSM80712, GSM80734, GSM80738, GSM80739, GSM80759, GSM80792, GSM80824, GSM80826, GSM80867, GSM80869, GSM95497, GSM95500, GSM97798, GSM97814
