# Supplementary material for: Cancer-testis antigen cyclin A1 is broadly expressed in ovarian cancer and is associated with prolonged time to tumor progression after platinum-based therapy
Source: BMC Cancer. 2015 Oct 24;15:784. doi: 10.1186/s12885-015-1824-6 (PMC4619521; doi:10.1186/s12885-015-1824-6)
Supplement: Additional file 2: — Table S1. Cox regression analysis for TTP and OS. Covariates other than Cyclin A1 staining intensity and percentage of Cyclin A1-positive cells for univariate analysis were chosen based on clinical relevance as described in earlier studies [9, 41, 42]. The four covariates with the highest p-values were analyzed in a multivariate Cox regression analysis [the number of 49 (TTP) and 41 (OS) events allows a maximum of four covariates]. bi: regression coefficient, CI: confidence interval, HR: hazard ratio. (DOCX 14 kb) [file 12885_2015_1824_MOESM2_ESM.docx]

Additional file 1: Table S1. Univariate and multivariate survival analysis (A) Time to progression, (B) overall survival

A

| Number of events: 49 | Univariate (log rank) | Multivariate (cox regression) | | |
| --- | --- | --- | --- | --- |
| covariate* | *p*-value | HR [*exp(b_i_)*] | 95% CI | *p*-value |
| **Cyclin A1 staining intensity (high versus low)** | **0.018** | **2.025** | **1.051-3.901** | **0.035** |
| % Cyclin A1-positive cells (100% versus partial positivity) | 0.253 | 1.107 | 0.592-2.069 | 0.750 |
| Histological grade | 0.520 |  |  |  |
| FIGO stage | 0.314 | 1.610 | 0.750-3.456 | 0.222 |
| Peritoneal carcinomatosis | 0.131 | 2.016 | 0.615-6.614 | 0.247 |
| Residual tumor | 0.683 |  |  |  |
| Age | 0.864^a^ |  |  |  |

B

| Number of events: 41 | Univariate (log rank) | Multivariate (cox regression) | | |
| --- | --- | --- | --- | --- |
| Covariate* | *p*-value | HR [*exp(b_i_)*] | 95% CI | *p*-value |
| Cyclin A1 staining intensity (high versus low) | 0.155 | 1.101 | 0.510-2.376 | 0.806 |
| **% Cyclin A1-positive cells (100% versus partial positivity)** | **0.044** | 1.735 | 0.817-3.684 | 0.152 |
| Histological grade | 0.745 |  |  |  |
| FIGO stage | 0.678 | 0.818 | 0.345-1.941 | 0.649 |
| Peritoneal carcinomatosis | 0.685 |  |  |  |
| Residual tumor | 0.982 |  |  |  |
| Age | 0.163^a^ | 1.002 | 0.972-1.032 | 0.919^b^ |

* covariates other than Cyclin A1 staining intensity and percentage of Cyclin A1-positive

cells for univariate analysis were chosen based on clinical relevance as described in earlier studies [quelle, s. o.]. The four covariates with the highest *p*-value were analyzed in a multivariate cox regression analysis (the number of 49 (TTP) and 41 (OS) events allows a maximum of four covariates). ^a^: dichotomizes, ^b^: decades

*b_i_*: regression coefficient, CI: confidence interval, HR: hazard ratio
