# Supplementary figures and images for: Cancer-testis antigen cyclin A1 is broadly expressed in ovarian cancer and is associated with prolonged time to tumor progression after platinum-based therapy
Source: BMC Cancer. 2015 Oct 24;15:784. doi: 10.1186/s12885-015-1824-6 (PMC4619521; doi:10.1186/s12885-015-1824-6)

## Slide 1
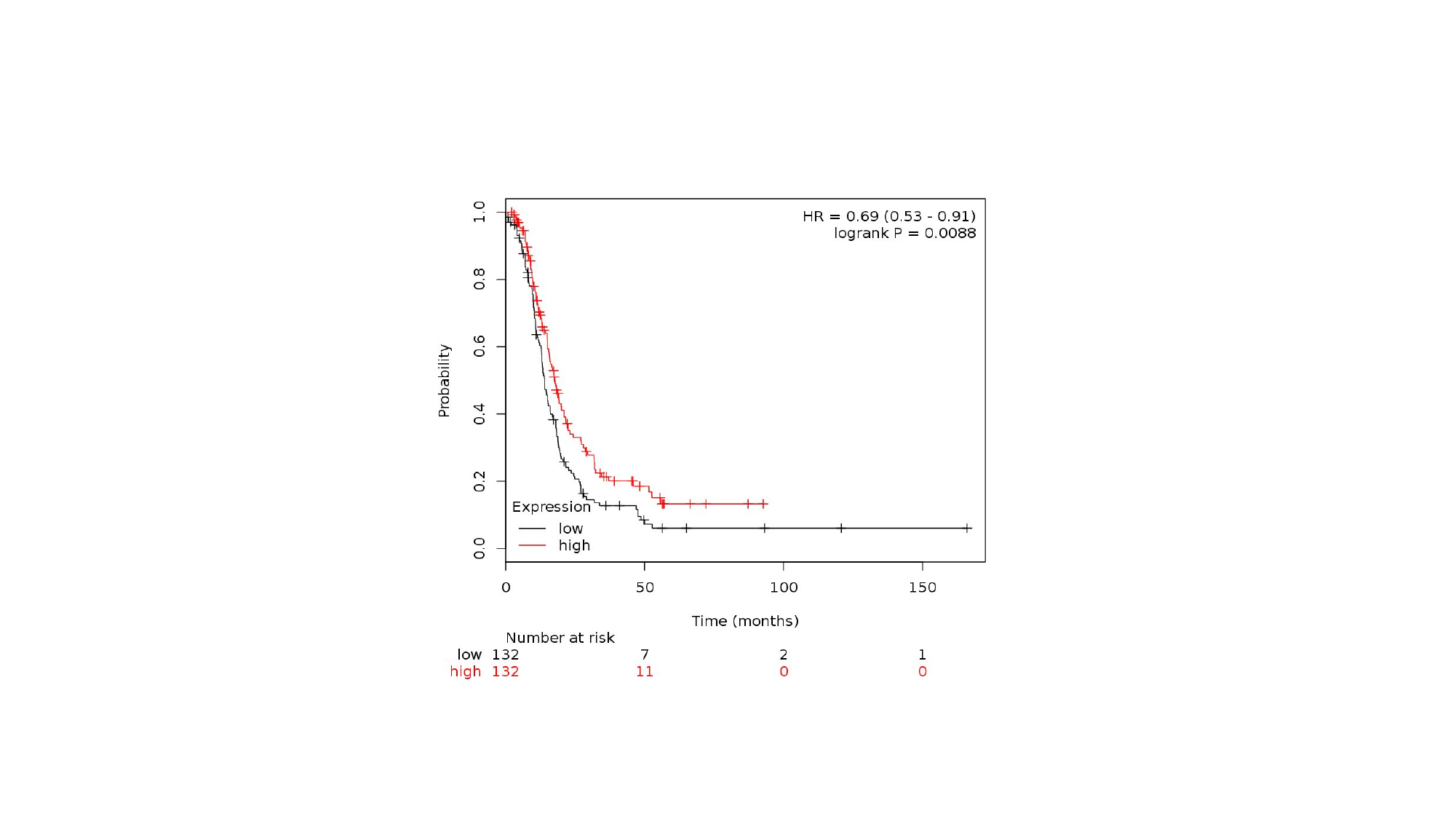

Supplement: Additional file 3: — Figure S1. Survival plot depicting the impact of Cyclin A1 expression (Affymetrix probe set 205899_at) on progression-free survival in the patient group with suboptimal debulking and platinum-based therapy using an online-accessible tool (www.kmplot.com/), database version 2015 [n = 1648]. Case selection [n = 264]: survival: PFS, split patients by median; restrictions: FIGO II, III, IV; histology: serous; debulk: suboptimal; chemotherapy: contains platinum. Log-rank p = 0.0088. (PPTX 84 kb) [file 12885_2015_1824_MOESM3_ESM.pptx]

## Slide 1
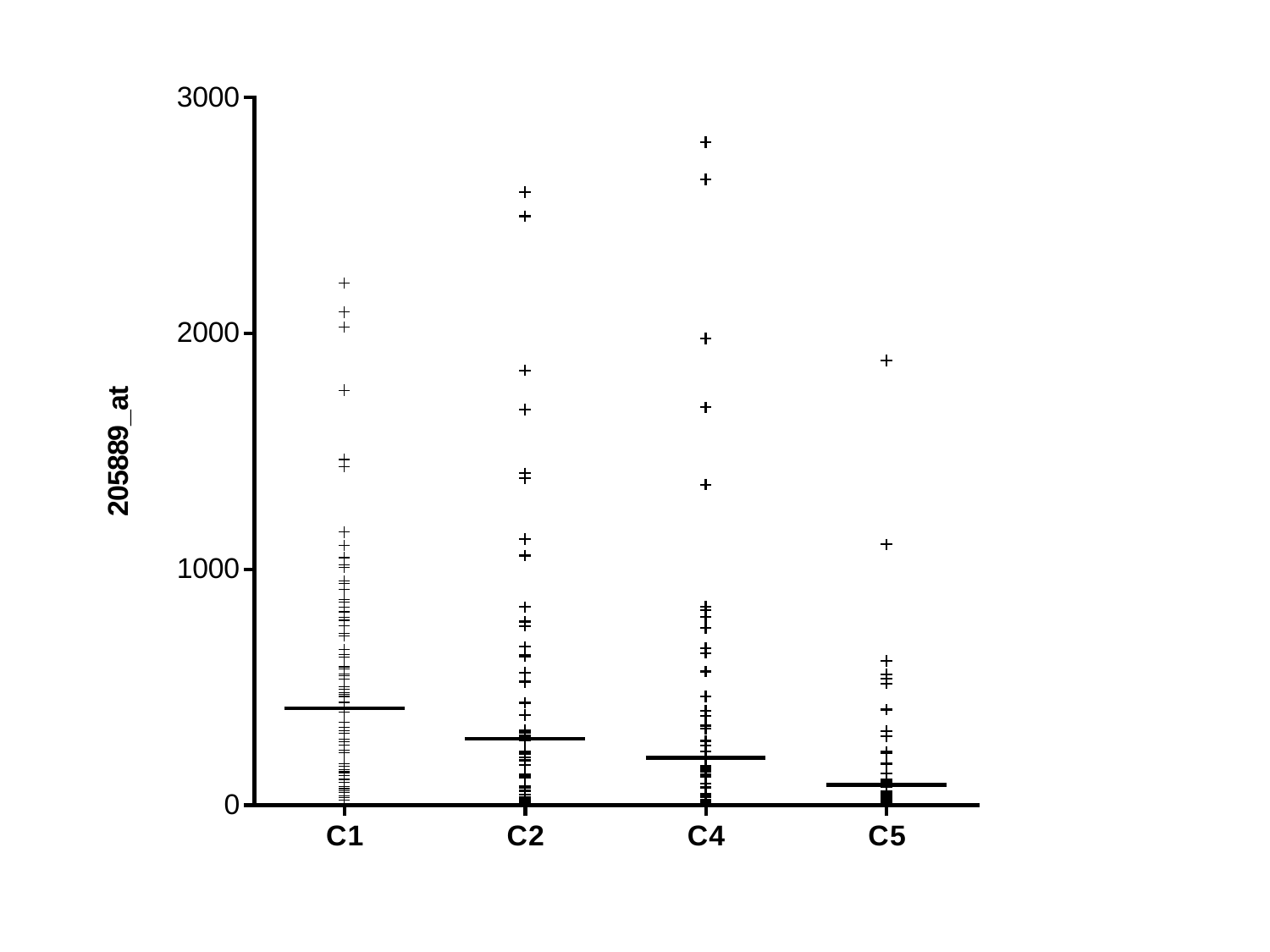

Supplement: Additional file 4: — Figure S2. Differences in Cyclin A1 expression between molecular tumor subtypes (1, 2, 4, and 5), according to the molecular classification of EOC by Tothill et al. Data sets were retrieved from the NCBI GEO database and normalized using the invariant set method (dChip 2.0 software) [23]. C1 showed significantly higher expression and C5 showed significantly lower expression (Kruskall Wallis; p = 0.001). Mean value + 3SD is marked by the horizontal bar. (PPT 147 kb) [file 12885_2015_1824_MOESM4_ESM.ppt]
